# Socioeconomic differentials in trends in the prevalence of hypertension and pre-hypertension and hypertension awareness, treatment, and control in rural Southwestern China

**DOI:** 10.1186/s12872-021-02062-x

**Published:** 2021-05-26

**Authors:** Lu-Ming Fan, Fang Wang, Min Zhao, Wen-Long Cui, Le Cai

**Affiliations:** 1 School of Public Health, Kunming Medical University, 1168 Yu Hua Street Chun Rong Road, Cheng Gong New City, 650500 Kunming China; 2 Department of Science and Technology, Kunming Medical University, 1168 Yu Hua Street Chun Rong Road, Cheng Gong New City, 650500 Kunming China

**Keywords:** Hypertension, Temporal trend, Socioeconomic differentials, Awareness, Treatment, Control, China

## Abstract

**Background:**

This study examines the socioeconomic differentials in trends in the prevalence of hypertension and pre-hypertension and hypertension awareness, treatment, and control in rural Southwestern China.

**Methods:**

Two cross-sectional interviews and health examination surveys were administered in rural Yunnan Province, including 6,350 consenting participants in 2009 and 6,359 consenting participants in 2016 (aged ≥ 35 years). Participant demographics, socioeconomic status (SES), and ethnicity, along with information about hypertension awareness, treatment, and control, were collected using similar questionnaires in the two surveys. The participants’ blood pressure levels were also measured.

**Results:**

From 2009 to 2016, the prevalence of hypertension substantially increased from 28.4% to 39.5% (*P* < 0.01), and awareness and control rose from 42.2 and 25.8% to 53.1 (*P* < 0.01) and 30.6% (*P* < 0.05), respectively. Although people with a higher education level also had higher awareness and control rates than the lower education level ones, there were no conspicuous differences in the improvement of awareness and control between publics with different education levels over the 7 years studied. Increases were observed in both rates of awareness and control in people with a high level of income (*P* < 0.01). However, only the awareness rate increased in participants with a low level of income. Furthermore, the prevalence (*P* < 0.01) and treatment (*P* < 0.05) of hypertension were higher in the Han people than in ethnic minorities.

**Conclusions:**

Individual SES has clear associations with trends in the prevalence, awareness, and control of hypertension. Future interventions to improve hypertension prevention and control should be tailored to address individual SES.

## Background

Hypertension is a widespread and severe public health issue throughout the world. It is also a major independent, progressive hazard for chronic non-communicable diseases (NCDs), specifically for cardiovascular diseases (CVD) with substantial economic and health losses across the globe [1]. The growing prevalence of hypertension can be observed in countries of all income levels [2]. Globally, the prevalence of hypertension increased from 25.9% in 2000 to 31.1% in 2010 [3]. Pre-hypertension, a medium phase between normal blood pressure (BP) and hypertension, is also common in the world and varies by race and region [4]. Studies have shown that individuals with pre-hypertension, particularly the elderly, are prone to developing hypertension [5, 6]. In China, the state hypertension prevalence in people aged ≥ 18 years was 25.2% in 2016, a dramatically increase compared to the prevalence of 5.1% in 1959, 7.7% in 1980, 13.6% in 1991, and 17.6% in 2002 [7].

The global hypertension awareness, treatment, and control increased from 41.4%, 31.8% and 11.7% in 2000 to 46.5%, 36.9% and 13.8% in 2010 [3]. Generally, the rates in awareness, treatment, and control of hypertension are meaningfully higher in developed countries than in developing ones [8, 9]. China has improved hypertension management substantially over the past years. Nevertheless, the rates in awareness, treatment and control for hypertension are remain relatively low (28.9%, 35.3%, and 3.4%, respectively) related to those described in developed countries [10, 11]. Awareness and knowledge of pre-hypertension and hypertension are critical for the prevention and reduction of hypertension, particularly in rural regions where the levels of awareness and treatment are relatively lower [12].

Socioeconomic status (SES) has been found to be related to pre-hypertension and hypertension. Specifically, a lower SES is in connection with a higher risk of pre-hypertension and hypertension [13, 14], regardless of the indicators of SES that are used. However, little is known about the associations between SES and trends in prevalence, awareness, treatment, and control of hypertension all around the world, particularly in rural and ethnic minority areas in China.

China is a multi-ethnic state, in which 56 ethnicities were documented by nation. Yunnan Province was located in Southwestern China, with a population of 47.14 million in 2016. Twenty-five ethnicities dwelling in the area, and 15 are discovered only in Yunnan. The number of ethnic minorities accounts for one-third of the total population in this area. Previous study indicated hypertension prevalence, awareness, treatment, and control were associated with individual SES and varied by ethnic minority group in Yunnan [15]. However, SES differences in trends in hypertension prevalence, awareness, treatment, and control in this particular aim crowd have not been recognized. Hence, the purpose of our study was to uncover the trends in the change in hypertension and pre-hypertension prevalence, and in hypertension awareness, treatment, and control and to investigate their relationships with SES indicators among Southwestern China’s rural population (aged ≥ 35 years) from 2009 to 2016.

## Methods

### Study area and population

In 2009 and 2016, two community-based, cross-sectional interviews and examination surveys were administered in two rural regions of Yunnan, China. In 2009, we employed a four-stage stratified random sampling method to choose the participants. In phase 1, all of the counties in Yunnan Province were classified into 2 groups, high and low, on the basis of per-capita gross domestic product (GDP). We randomly chosen one county from each of those two groups, for add up to 2 counties. In phase 2, each chosen county was also divided into 3 categories based on per-capita GDP: advantaged, average, or disadvantaged. From each of these 3 categories, one township was randomly chosen, for 6 townships in total. In phase 3, three villages were selected by probability proportional to size (PPS) method from each township. In phase 4 and ending, from the administration in each chosen village, a residents’ list aged 35 years and over was procured. Then, a simple random sampling method was employed to choose eligible persons from each chosen village. In 2016, we employed a uniform four-stage stratified random sampling technique to enable us to choose participants from the two rural regions.

### Data collection and measurement

Similar pre-tested questionnaires were employed to assemble data from participants in the two surveys [16]. Both in 2009 and 2016, face-to-face interviews were conducted to each consenting participant by trained interviewers. Information about participants’ demographic characteristics (sex, age, ethnicity, education, and annual household income), diagnosis, treatment, and control of hypertension were converged. The measured BP was documented accompanied by those from the questionnaire.

BP was measured by trained interviewers using standardized mercury sphygmomanometers in 2009 and 2016, and taken from the individuals’ right arm after they rested for as a minimum 5 min in the sitting situation and had not eaten, smoked, or exercised. Three successive BP measurements were obtained following American Heart Association (AHA) recommendations [17]. In our study, we took the mean BP of three readings. To sustain the accuracy of BP results taken by mercury sphygmomanometer, an oscillometer device was used once a week.

### Ethics approval

The Ethics Committee at Kunming Medical University authorized our study ahead of the start of the research.

### Definition

Based on recommendations in the eighth Report of the Joint National Committee on the Prevention, Detection, Evaluation, and Treatment of High Blood Pressure (JNC 8) [18], pre-hypertension was defined in our study as a systolic blood pressure (SBP) of 120–139 mmHg, and/or diastolic blood pressure (DBP) of 80–89 mmHg. Hypertension was established when a person who has a mean SBP ≥ 140 mmHg, DBP ≥ 90 mmHg, and/or currently being on antihypertensive therapy. A former diagnosis of hypertension by a qualified medical institution was also viewed hypertension in our study.

Among individuals with hypertension, awareness was established in our study when an individual had reported a former hypertension diagnosis in a health facility. Treatment was established when antihypertensive therapy in the last 14 days was reported by participants. Moreover, control was established when a person who has a mean SBP < 140 mmHg and DBP < 90 mmHg posttreatment.

Ethnicity was derived from China’s 56 state-recognized ethnicities, and was then classified into two groups in our study: Han Chinese or ethnic minority. Illiterate was defined as a person who has no capability to read with grasp or to write simple sentences. The level of education was then divided into two sets: illiterate and primary (grades 1–6) or higher. Approximate yearly household income was separated into two classifications (low and high), with the median value as the cut off point. Low referred to an approximate yearly household income < $945, and high referred to a yearly household income ≥ $945.

### Statistical analysis

We employed SPSS 22.0 software to analyze all data double-entered into an EpiData 3.1. Descriptive analysis methods and the chi-squared test and t-test were computed in this study. The mean values of SBP and DBP were stated as the mean ± standard deviation ($\overline{x}$ ± *s*). Counts and percentages were employed to express categorical variables in the study. A chi-squared test was applied to make a comparison on categorical variables between survey years and genders, and the t-test was analyzed continuous variables. We have adjusted the prevalence of hypertension and pre-hypertension, the rates in awareness, treatment, and control of hypertension, with age using a direct standardization technique for the 2010 population aged 35 years and over in China. All of the statistical significance determinations were grounded in two-tailed *P* values of < 0.05 in the study.

## Results

In 2009 and 2016, a total of 6,600 people aged ≥ 35 years were invited in two surveys, of which 6,350 in 2009 and 6,359 in 2016 agreed to participate, yielding a total response rate of 96.2% and 96.3%, respectively.

Table 1 indicates the general features of the participants. Among the study participants in 2009, 48.2% were male and 51.8% were female. In 2016, 49.4% were male and 50.6% were female. There were no valid changes in proportion of males (*P* = 0.204), mean age (*P* = 0.154), and proportion of people with a low approximate yearly household income (*P* = 0.128) between the two survey years. Nevertheless, the rate of illiteracy for females shortened from 50.9% in 2009 to 38.7% in 2016 (*P* = 0.001). Females had a higher illiteracy rate (*P* = 0.015) and a higher low approximate yearly household income rate (*P* = 0.023) than males both in 2009 and 2016.

**Table 1:** General characteristics of the study population by survey year

Characteristics	Survey year					
	2009			2016		
	Male (n = 3067)	Female (n = 3283)	All (n = 6350)	Male (n = 3143)	Female (n = 3216)	All (n = 6359)
Age						
35–44 years	712 (23.2)	761 (23.2)	1473 (23.2)	737 (23.4)	714 (22.2)	1451 (22.8)
45–54 years	764 (24.9)	813 (24.8)	1577 (24.8)	830 (26.4)	895 (27.8)	1725 (27.1)
55–64 years	780 (25.4)	838 (25.5)	1618 (25.5)	740 (23.5)	839 (26.1)	1579 (24.8)
≥ 65 years	811 (26.4)	871 (26.5)	1682 (26.5)	836 (26.6)	768 (23.9)	1604 (25.2)
Approximate yearly household income (%)						
Low	1230 (40.1)	1768 (53.9)*	2998 (47.2)	1264 (40.2)	1614 (50.2)*	2878 (45.3)
High	1837 (59.9)	1515 (46.1)	3352 (52.8)	1879 (59.8)	1602 (49.8)	3481 (54.7)
Level of education (%)						
Illiterate	740 (24.1)	1671 (50.9)**	2411 (38.0)	856 (27.2)	1244 (38.7)**	2100 (33.0)
Primary (grade 1–6) or higher	2327 (75.9)	1612 (49.1)	3939 (62.0)	2287 (72.8)	1972 (61.3)	4259 (67.0)

* *P* < 0.05, ** *P* < 0.01

Table 2 presents the prevalence of pre-hypertension and hypertension in two survey years and SES in rural Yunnan. Over the 7 years studied, from 2009 to 2016, although the prevalence of pre-hypertension did not differ, the hypertension prevalence boosted from 28.4 to 39.5% (*P* = 0.001), and among the subcategories, the increasing rates existed in sex, ethnicity, level of education, and approximate yearly household income. The prevalence of hypertension in males and in females increased from 27.6% and 28.8% to 39.5% and 39.8%, respectively (*P* = 0.001 and *P* = 0.001), and in the Han majority population, it rose from 27.9% to 40.3% (*P* = 0.001). Moreover, in the illiterate population, it increased from 26.5% to 54.7% (*P* = 0.001), and in participants with a low approximate yearly household income, it increased from 28.5 to 48.6% (*P* = 0.001). In both 2009 and 2016, the prevalence of pre-hypertension was higher in male participants than in female individuals (*P* = 0.012). In 2016, the hypertension prevalence was higher in Han majority population than in ethnicity minority population (*P* = 0.001), as well as in illiterate population than in their counterparts (*P* = 0.001). Moreover, the hypertension prevalence was higher in individuals with a low approximate yearly household income than in participants with a high approximate yearly household income (*P* = 0.001).

**Table 2:** Age-standardized prevalence of pre-hypertension and hypertension by survey year and socioeconomic status in rural Yunnan Province, China

Characteristics	2009		2016	
	Pre-hypertension n (%)	Hypertension n (%)	Pre-hypertension n (%)	Hypertension n (%)
Sex				
Male	1569 (52.1)	816 (27.6)	1676 (53.5)	1242 (39.5)**
Female	1444 (43.7)**	975 (28.8)	1504 (46.4)**	1280 (39.8)**
Age				
35–44 years	663 (45.2)	170 (11.7)	705 (48.2)	288 (19.5)
45–54 years	733 (46.4)	408 (25.3)	862 (49.7)	699 (34.2)
55–64 years	776 (47.7)	530 (31.9)	795 (50.1)	745 (40.8)
≥ 65 years	841 (50.3)**	683 (40.8)**	818 (51.6)*	790 (45.9)**
Ethnicity				
Han	2796 (47.3)	1660 (27.9)	3003 (48.6)	2417 (40.3)**
Minority	217 (46.4)	131 (28.3)	177 (48.3)	105 (27.2)**
Level of education				
Illiterate	1073 (46.8)	649 (26.5)	1053 (50.3)	1145 (54.7)**
Primary (grade 1–6) or higher	1940 (47.7)	1142 (29.6)	2127 (49.7)	1377 (32.0)**
Approximate yearly household income				
Low	1367 (46.9)	861 (28.5)	1422 (49.8)	1406 (48.6)**
High	1646 (47.8)	930 (27.8)	1758 (50.1)	1116 (32.4)**
All	3013 (47.2)	1791 (28.4)	3180 (50.0)	2522 (39.5)**

* *P* < 0.05, ** *P* < 0.01

Table 3 shows the hypertension awareness, treatment, and control in two study years and SES in rural areas of Yunnan, China. The hypertension awareness increased from 42.2 to 53.1% (*P* = 0.001), and control rose from 25.8 to 30.6% (*P* = 0.026) over the 7 years studied. We found the trends of increase both in males and females, however, only observed in Han majority population and in people with a high level of income. Additionally, the awareness of hypertension also increased in those behind a low level of income. Although the hypertension awareness and control were higher in people who have a higher position in education level than in their counterparts, the improvement in awareness and control did not differ between groups of different education levels over the seven years studied. Further, there was no noteworthy change in treatment among the subcategories layered by sex (*P* = 0.272), education (*P* = 0.603), and income (*P* = 0.358) between in 2009 and in 2016. In both 2009 and 2016, the hypertension awareness and control were higher in females than in males (*P* = 0.017 and *P* = 0.033), and the ethnicity minority population had comparatively lower treatment rates than the Han people (*P* = 0.014).

**Table 3:** Age-standardized awareness, treatment, and control of hypertension by survey year and socioeconomic status in rural Yunnan Province, China

Characteristic	2009			2016		
	Awareness of condition n (%)	Under treatment n (%)	Condition controlled n (%)	Awareness of condition n (%)	Under treatment n (%)	Condition controlled n (%)
Sex						
Male	304 (37.3)	273 (74.2)	79 (21.5)	619 (49.8)**	443 (73.6)	182 (29.4)**
Female	452 (46.4)**	444 (79.0)	161 (28.6)*	720 (56.3)**	571 (77.3)	228 (31.7)*
Age						
35–44 years	35 (20.6)	27 (60.0)	16 (35.6)	84 (29.2)	73 (70.0)	25 (29.8)
45–54 years	141 (34.6)	128 (73.6)	43 (25.7)	276 (46.1)	199 (72.1)	85 (30.8)
55–64 years	239 (45.1)	222 (75.5)	75 (25.3)	404 (54.2)	304 (75.7)	111 (27.5)
≥ 65 years	341 (49.9)**	340 (80.8)**	106 (25.2)	575 (64.6)**	458 (80.2)**	189 (32.9)
Ethnicity						
Han	709 (42.7)	674 (79.7)	226 (25.7)	1287 (53.2)*	981 (76.2)	394 (31.2)*
Minority	47 (35.9)	43 (64.3)*	14 (27.5)	52 (49.5)	33 (63.5)*	16 (30.0)
Level of education						
Illiterate	280 (40.1)	255 (75.3)	96 (24.0)	561 (49.0)	427 (76.1)	129 (23.0)
Primary (grade 1–6) or higher	476 (44.7)*	462 (75.0)	144 (29.1)*	778 (56.5)**	587 (75.4)	281 (36.1)**
Approximate yearly household income						
Low	372 (43.2)	341 (75.0)	125 (27.1)	743 (52.8)**	427 (76.1)	194 (26.1)
High	384 (41.3)	376 (75.5)	115 (24.7)	596 (53.4)**	587 (75.4)	216 (36.2)**
All	756 (42.2)	717 (75.1)	240 (25.8)	1339 (53.1)**	1014 (75.7)	410 (30.6)*

* *P* < 0.05, ** *P* < 0.01

## Discussion

The findings indicated high rates in the prevalence of pre-hypertension and hypertension, relatively low levels in hypertension awareness, treatment, and control, and a total increase in the hypertension prevalence, awareness, and control in rural Southwestern China over the seven years studied. Moreover, the findings also indicated that ethnicity and individual SES have significant relationships with actual hypertension prevalence, awareness, and control, as well as the temporal trends in the hypertension prevalence, awareness, and control.

In the study, the prevalence of pre-hypertension (50.0%) in Yunnan Province was greater than that observed in China and other Asian and low- and middle-income countries [19–23]. The study did not show a growth in the prevalence of pre-hypertension over the 7 years studied. However, it revealed a substantially increasing trend in hypertension prevalence and indicated that hypertension is a chief and increasing public health issue in the regions under study. This possibly results from increasing prevalence rates of obesity (from 6.7% in 2009 to 9.0% in 2016), central obesity (from 43.5% in 2009 to 51.1% in 2016), and diabetes (from 7.7% in 2009 to 9.5% in 2016) in the regions under study, and obesity, central obesity, and diabetes are important contributors to hypertension. The findings suggested that more effective interventions are needed to avoid and control hypertension. Encouragingly, the hypertension awareness, treatment, and control rates (53.1%, 75.7%, and 30.6%, respectively) in 2016 were higher than those found in a national study of China (36.0%, 22.9%, and 5.7%) [24], indicating that hypertension health education programs and improving access to healthcare have helped achieve progress in rural Southwestern China.

The study also showed ethnic differences in temporal trends in hypertension prevalence, and hypertension awareness, treatment, and control in rural Southwestern China. Specifically, the Han people had higher prevalence of hypertension than ethnic minority populations over the seven years studied. One of the reasons might be that ethnic minorities have healthier lifestyles, such as plain food and more activities [25]. In both 2009 and 2016, the treatment rate of hypertension in ethnic minority populations was significantly lower than which in the Han people, which may be attributed to the economically disadvantaged status of the ethnic minority population [26]. Furthermore, only the Han majority population improved the rates in hypertension awareness and control. Thus, specific efforts must be taken in the ethnic minority groups to advance the awareness, treatment, and control of hypertension as well as to improve their economic status and their access to medical services.

In the study population, less educated and low-income individuals were more likely to suffer from hypertension than their more educated and high-income counterparts. This result is consistent with that from previous studies [14, 15, 27]. This possibly due to the fact that less educated individuals had higher prevalence of obesity, central obesity, and diabetes than their more educated counterparts (13.0% vs. 7.0%, 56.4% vs. 43.5%, and 9.7% vs. 6.5%, *P* < 0.05), while prevalence of obesity and central obesity was higher in low-income individuals than in high-income counterparts (9.5% vs. 8.6% and 51.1% vs. 37.3%, *P* < 0.05) in this study, and diabetes and being obese or central obese are well recognized established major risk factors for CVD in previous studies. Furthermore, over the seven years we studied, the hypertension prevalence markedly increased in both less educated and low-income people. The interactions of SES and multiple CVD risk factors thereby underscore an urgent need for improving community-based hypertension prevention strategies focused particularly on individuals with low levels of education and income.

In this study, higher rates in awareness and control were found in more educated people than less educated participants in both 2009 and 2016. However, there was no remarkable change in the increase of awareness, treatment, and control between different education levels groups. In particular, the study showed stable treatment rates of hypertension between the two survey years, suggesting that the treatment of hypertension had entered a bottleneck stage in the study region, and novel effective measures should be taken to continue to improve the treatment rate. Although the treatment of hypertension did not differ in groups with diverse education level, the control of hypertension was significantly lower in illiterate groups than in educated groups. The specific reasons need further study.

Although there was a significant increase in awareness between different income groups over the 7 years studied, it only appeared in the high-income group for control. Encouragingly, the study revealed a similar treatment rate of hypertension between low-income participants and their higher-income counterparts both in 2009 and in 2016. Although the low-income participants had equivalent awareness and treatment of hypertension as their higher-income counterparts, the rate of control was dramatically lower in the low-income participants than their counterparts over the 7 years studied. The reasons might be low-income people use incorrect treatment methods or do not adhere to their treatment for a long time [28]. The findings underline an urgent need to improve the control of hypertension for the low-income population.

There were some limitations in the study. First, as the data were collected using a cross-sectional design, we were unable to determine causal relationships. Second, the trends in prevalence, awareness, treatment, and control of hypertension were analyzed based on a seven years study period, which is a relatively short-range time period; to determine whether the trends will persist over a longer time frame, more data will be needed. Third, the study lacked lipid, diet, alcohol, and physical inactivity -related data, and lipid, diet, alcohol and physical inactivity may be important factors influencing hypertension. So the full range of risk factors attributable to hypertension was not captured. Further research is needed to examine the contribution of other risk factors to hypertension in rural southwestern China. Fourth, due to the lack of data on pharmacological treatment/care for multiple cardiovascular risk factors, we did not analyze their interactions with SES.

## Conclusions

In conclusion, the study displayed that the prevalence of hypertension as well as hypertension awareness and control significantly increased in rural Southwestern China from 2009 to 2016, with ethnicity and individual SES having clear associations with the trends in prevalence, awareness, and control of hypertension. However, the hypertension treatment did not differ over the 7 years studied. The findings indicated that the policymakers should increase levels of awareness and control to promote the hypertension prevention and management, as well as employ effective interventions that consider socioeconomic disparities.

## Data Availability

The datasets used and/or analyzed during the current study is available from the corresponding author on reasonable request.

## Abbreviations

AHA: American Heart Association;
BP: Blood pressure;
CVD: Cardiovascular diseases;
DBP: Diastolic blood pressure;
GDP: Gross domestic product;
NCDs: Non-communicable diseases;
PPS: Probability proportional to size;
SBP: Systolic blood pressure;
SES: Socioeconomic status
